# Supplementary figures and images for: Improving lysosomal ferroptosis with NMN administration protects against heart failure
Source: Life Sci Alliance. 2023 Oct 4;6(12):e202302116. doi: 10.26508/lsa.202302116 (PMC10551641; doi:10.26508/lsa.202302116)

Fig 2

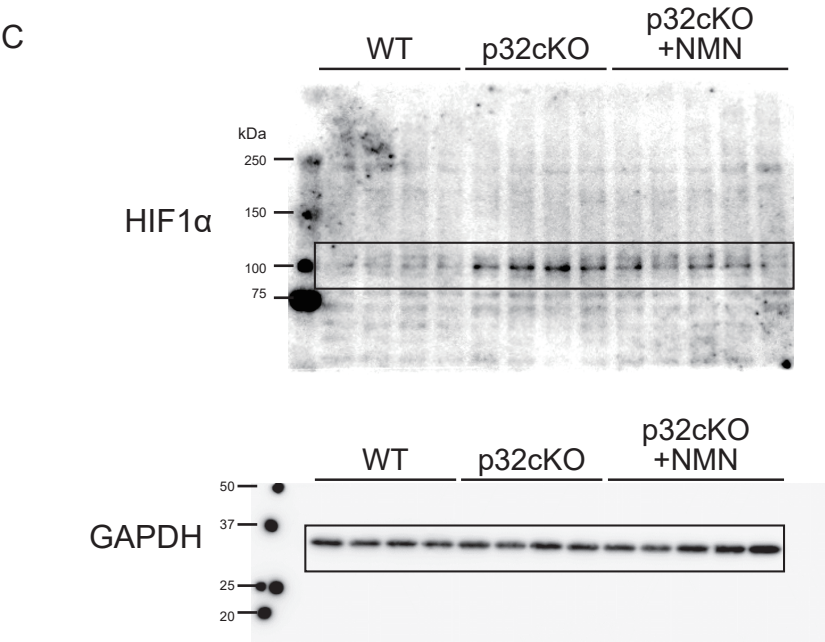

Supplement: Supplementary file 1 [file LSA-2023-02116_SdataF2.pdf]

Figure 4

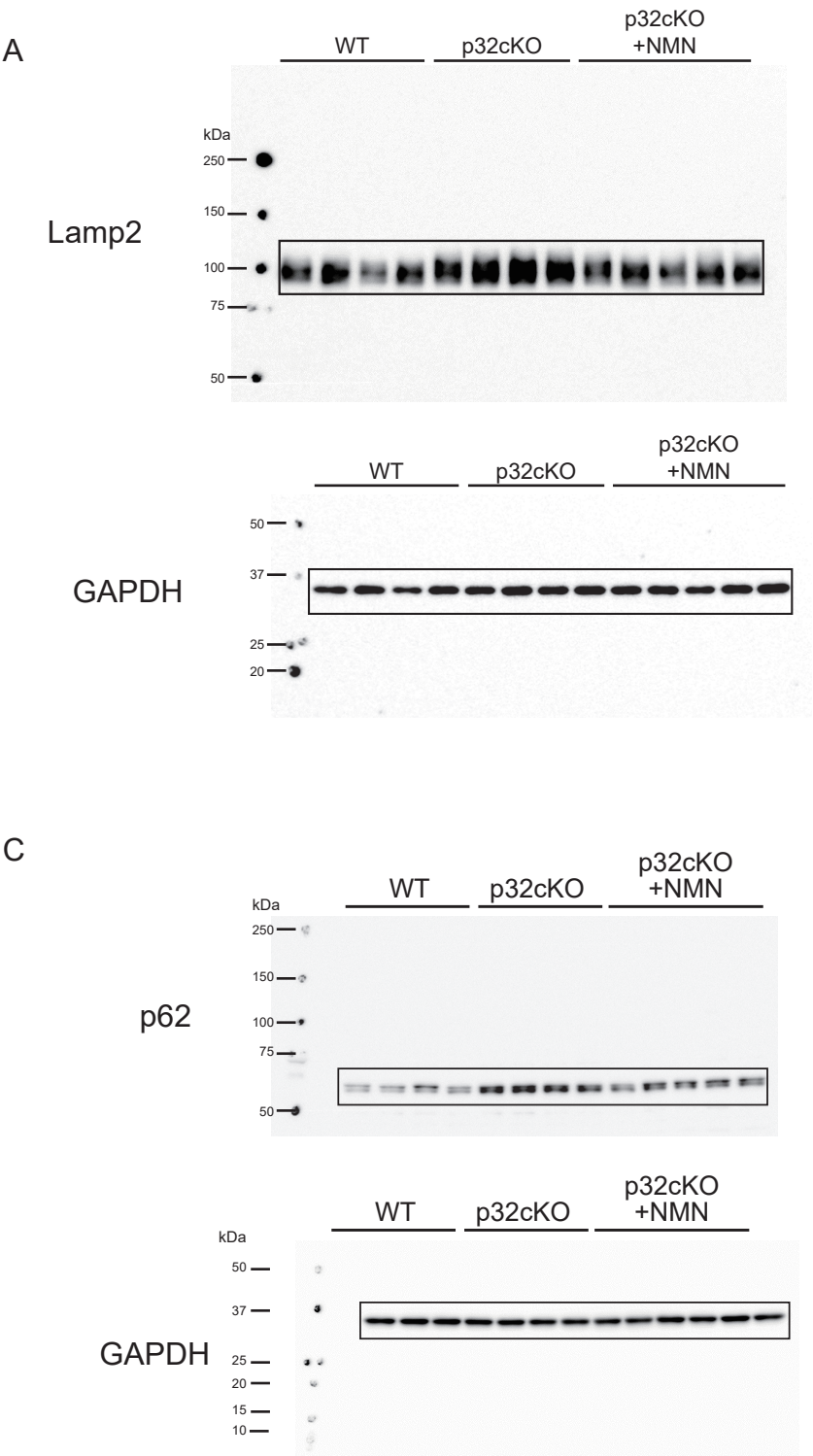

Supplement: Supplementary file 2 [file LSA-2023-02116_SdataF4.pdf]

Figure 5

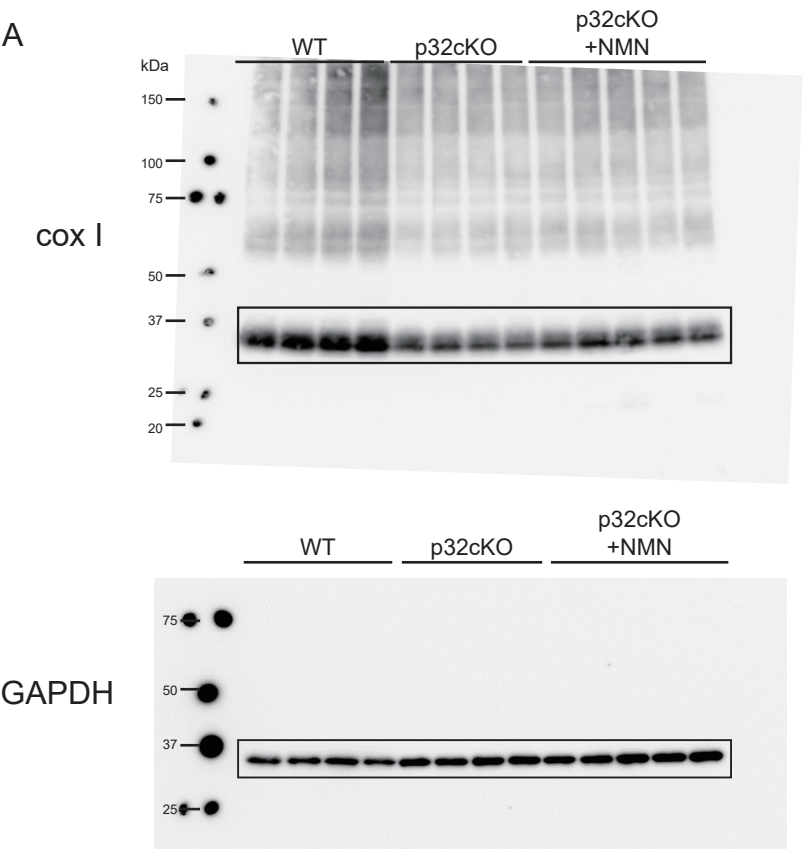

Supplement: Supplementary file 3 [file LSA-2023-02116_SdataF5.pdf]

Figure 6

D

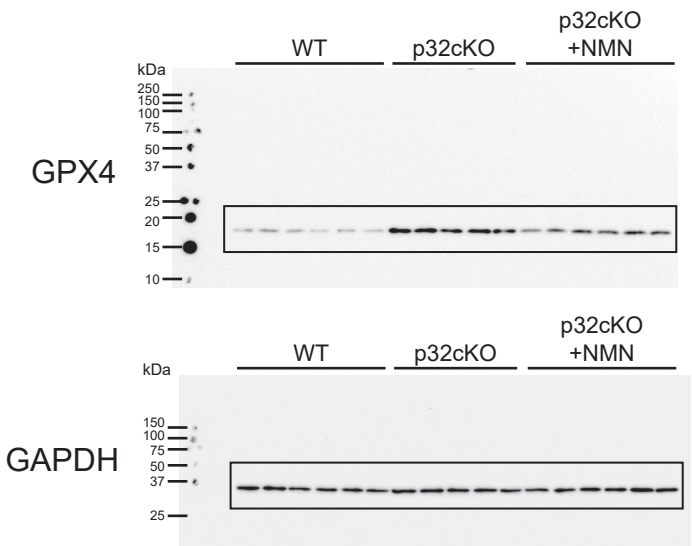

H

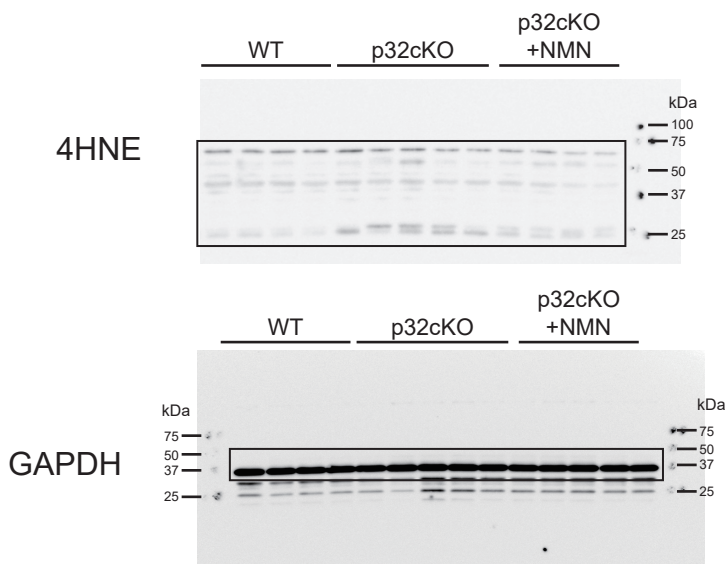

Supplement: Supplementary file 4 [file LSA-2023-02116_SdataF6.pdf]

Supplementary Fig. S3

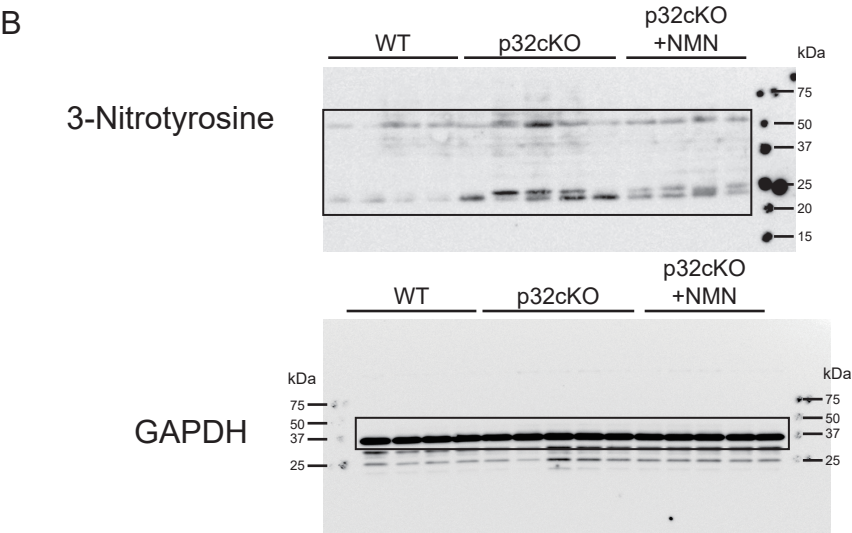

Supplement: Supplementary file 5 [file LSA-2023-02116_SdataFS3.pdf]
