# Supplementary material for: Improving lysosomal ferroptosis with NMN administration protects against heart failure
Source: Life Sci Alliance. 2023 Oct 4;6(12):e202302116. doi: 10.26508/lsa.202302116 (PMC10551641; doi:10.26508/lsa.202302116)
Supplement: Supplementary file 6 [file LSA-2023-02116_TableS1.docx]

| Target | Forward (5'->3') | Reverse (5'->3') |
| --- | --- | --- |
| Nampt | tacagtggccacaaattcca | caattcccgccacagtatct |
| Nmnat1 | gaagtgggctgatcaaaagc | ccagcccgagtgatacagat |
| Nmnat3 | tccagcagtttcagcacaac | gaggccctctagccagtctt |
| Sirt3 | tcctcgaaggaaagatgtgg | gcatgaagtcttgctggaca |
| Tfeb | tcctctggcggcagtactat | gtgccatctagtcccaggaa |
| Fgf21 | gggaggatggaacagtggta | gtcctccagcagcagttctc |
| Gdf15 | cttgaagacttgggctggag | taagaaccaccggggtgtag |
| Atf4 | tcgatgctctgtttcgaatg | agaatgtaaagggggcaacc |
| Ptgs2 | agaaggaaatggctgcagaa | gctcggcttccagtattgag |
| Acsl4 | ttggtcagggatatgggctg | gccaccgatcacaatctcac |
| ANF | catcaccctgggcttcttcct | tgggctccaatcctgtcaatc |
| β-MHC | atgtgccggaccttggaa | cctcgggttagctgagagatca |
| Glud1 | gggatttggtaatgtgggcc | agtatgtcacagtcggcctc |
| Gpx4 | agtacaggggtttcgtgtgc | ggctgcaaactccttgattt |
| Nrf2 | ggtccacatttccttcatgg | ccgtccaggagttcagagag |
| Chac1 | ataccaagttcgaggggagc | tctgtgtggcaatgacctct |
| Ho1 | taagctggtgatggcttcct | cctgagaggtcacccaggta |
| Psap | tggaccagtattccgaggtc | tctgctcataggggtccatc |
| Slc7a11 | cccagatatgcatcgtcctt | cgtctgaaccacttgggttt |
| FTH1 | cgagatgatgtggctctgaa | gtgcacactccattgcattc |
| Mfrn2 | cctatcagccccctgtacg | gcctgttgctgtgacgttc |
| 18s | cgcggttctattttgttggt | agtcggcatcgtttatggtc |

Supplementary Table 1 List of primer used in this study
